# Long-term effectiveness of tonsillotomy versus tonsillectomy: A 12-year follow-up study

**DOI:** 10.1007/s00405-024-09000-5

**Published:** 2024-10-01

**Authors:** Julia Virkkunen, Johanna Nokso-Koivisto, Anniina J. Sakki

**Affiliations:** Department of Otorhinolaryngology, Head and Neck Surgery, Helsinki University Hospital and University of Helsinki, Helsinki, Finland

**Keywords:** Tonsillotomy, Partial tonsillectomy, Intracapsular tonsillectomy, Disease-specific quality of life

## Abstract

**Purpose:**

To evaluate the long-term effectiveness of tonsillotomy (TT) compared to tonsillectomy (TE) with respect to disease-specific quality of life (QOL), sleep-disordered breathing symptoms, throat infections, and rate of reoperations over a median follow-up period of 12 years.

**Methods:**

All patients < 16 years of age who underwent tonsil surgery between 2010 and 2011 at Helsinki University Hospital, Finland, were included in the study. In 2023, the patients answered a questionnaire concerning tonsil-related issues and a modified Tonsil and Adenoid Health Status Instrument (disease-specific QOL). Information about possible revisits and reoperations was gathered.

**Results:**

The study population consisted of 189 respondents, of which 87 had undergone TT and 102 TE. The median follow-up was 11.8 years in the TT group and 12.4 years in the TE group. The disease-specific QOL was equally good in both groups. Throat infections had not been an issue for the vast majority of patients and occurred to the same extent after TT and TE. The majority of participants (79.0% TT, 86.9% TE) were satisfied with the surgery, with no significant differences between the groups. After TT and TE, there were only a few revisits due to tonsil-related problems, and the reoperation rate was 6.9% and 1.0%, respectively.

**Conclusion:**

Long-term clinical effectiveness of TT seems excellent. Compared to TE, with TT, equal disease-specific QOL can be achieved with a less invasive surgical method. Over a median follow-up period of 12-years, TT was not shown to be associated with an increased risk of tonsil infection problems.

## Introduction

Both indications and surgical techniques of tonsil surgery have undergone several changes over the past centuries. To decrease postoperative sequela and complications, partial tonsillectomy or tonsillotomy (TT) was reintroduced in the late twentieth century [1, 2]. TT is currently the most popular surgical method in several countries in the treatment of sleep-disordered breathing (SDB) due to tonsillar hyperplasia in children [3–6].

In comparison to tonsillectomy (TE), TT has been associated with a lower risk of post-operative haemorrhage, decreased postoperative pain, and a faster return to normal diet and activities [7–11]. Tonsil-related symptoms are known to lower quality of life both in children and adults [12, 13], and TT has been shown to improve quality of life in children suffering from SDB [14, 15]. However, there is a risk of tonsillar regrowth after TT, and questions have been raised regarding whether tonsillar remnants may later lead to recurrent or chronic tonsilitis, or formation of peritonsillar abscesses [16, 17].

The majority of previous TT studies have focused on short-term outcomes after surgery, and long-term studies of TT are scarce [18, 19]. The aim of this study was to evaluate the long-term effectiveness of TT compared to TE with respect to disease-specific quality of life (QOL), SDB symptoms, throat infections, and rate of reoperations over a follow-up period of 12 years. To date, this study presents the longest follow-up period after TT ever considered.

## Methods

### Study design

This was a retrospective cohort and survey study. The study population consisted of all children under the age of 16 years who underwent tonsil surgery at the Department of Otorhinolaryngology, Helsinki University Hospital, Helsinki, Finland, between 1.1.2010 and 31.12.2011. Patient data were gathered from the patient database with procedure codes (Nordic Medico-Statistical Committee Classification of Surgical Procedures) EMB10 (TE), EMB15 (TT), and EMB20 (adenotonsillectomy). Children who underwent concomitant adenoidectomy were included in the study.

Possible revisits to the study hospital between 1.1.2010 and 31.3.2023 after the initial operation due to tonsil or throat-related problems were gathered from patient records. Of these, visits due to immediate postoperative complications during the first postoperative month were excluded. Information about possible reoperations was also collected.

The survey regarding tonsil-related history and symptoms was built and data compiled using the Research Electronic Data Capture (REDCap) tool (version 14.0.20, Vanderbilt University, Nashville, USA) hosted on the University of Helsinki’s servers [20, 21]. Recruitment letters with instructions for the electronic questionnaire were sent to the study patients in April–May 2023. The questionnaire was closed at the end of October 2023.

### Surgery

The technique for TT was adopted from Hultcrantz et al. [22]. TTs were performed using a BM-780 II radiofrequency generator (Sutter Medizintechnik GmbH, Freiburg, Germany) and a bent needle electrode (ARROWtip, Re-Usable, Sutter Medizintechnik GmbH, Freiburg, Germany). Only the protruding part of the tonsil was cut in the plane of the pharyngeal pillars. TEs were performed using either a combination technique (cold dissection with scissors and haemostasis obtained with bipolar diathermia) or a hot technique (dissection and haemostasis with bipolar diathermia). Surgeons were both residents and specialists in otolaryngology.

### Questionnaire

The Tonsil and Adenoid Health Status Instrument (TAHSI) was used to assess QOL after tonsil surgery. TAHSI is a disease-specific QOL instrument for tonsil and adenoid disease, validated for children between 2 and 16 years [23]. Witsell et al. modified TAHSI to be suitable for adults [24]. The adult TAHSI consists of 18 questions divided into six subscales: airway and breathing (questions 1, 7, 11), infection (questions 2, 8, 9, 16), health care utilization (questions 3, 4, 5, 6), eating and swallowing (questions 12, 13, 14, 15), cost of care (question 10), and breath (questions 17, 18) (Table 1). The adult TAHSI is not validated, but 11/18 of the questions are identical to the original validated version [23, 24]. To the best of our knowledge, there is no other validated disease-specific QOL instrument for adults that would cover both obstructive and infective symptoms of the tonsils. Because the vast majority of the participants in our study were older than 15 years, we decided to use the adult version of TAHSI by Witsell et al.

**Table 1:** Modified TAHSI questionnaire

Tonsil and Adenoid Health Status Instrument The following questions deal with problems that might be related to tonsils and adenoids. For each question, choose the option that best describes the severity of condition in the time frame asked. Please do not leave any questions unanswered Have any of the following conditions been a problem for you after the surgery? If so, how much of a problem?					
	Not a problem	Very mild problem	Moderate problem	Fairly bad problem	Severe problem
1. Snoring loudly during sleep					
a) During the past 6 months	0	1	2	3	4
b) At any point after the surgery except during the past 6 months	0	1	2	3	4
2. Strep throat infections					
a) During the past 6 months	0	1	2	3	4
b) At any point after the surgery except during the past 6 months	0	1	2	3	4
3. Many trips to the doctor’s office for a sore throat					
a) During the past 6 months	0	1	2	3	4
b) At any point after the surgery except during the past 6 months	0	1	2	3	4
4. Taking antibiotics for more than 3 weeks straight for a sore throat					
a) During the past 6 months	0	1	2	3	4
b) At any point after the surgery except during the past 6 months	0	1	2	3	4
5. Taking antibiotics over and over for less than 2 weeks at a time for a sore throat					
a) During the past 6 months	0	1	2	3	4
b) At any point after the surgery except during the past 6 months	0	1	2	3	4
6. Many calls to the doctor’s office for a sore throat					
a) During the past 6 months	0	1	2	3	4
b) At any point after the surgery except during the past 6 months	0	1	2	3	4
7. Irregular or stopped breathing, also known as apnoea, during sleep					
a) During the past 6 months	0	1	2	3	4
b) At any point after the surgery except during the past 6 months	0	1	2	3	4
8. Repeated short-term (or acute) infections of the tonsils that last less than 2 weeks					
a) During the past 6 months	0	1	2	3	4
b) At any point after the surgery except during the past 6 months	0	1	2	3	4
9. Constant, or chronic, infection of the tonsils that lasts more than 2 weeks					
a) During the past 6 months	0	1	2	3	4
b) At any point after the surgery except during the past 6 months	0	1	2	3	4
10. The cost of medical care and prescription					
a) During the past 6 months	0	1	2	3	4
b) At any point after the surgery except during the past 6 months	0	1	2	3	4
11. Breathing through the mouth during the day					
a) During the past 6 months	0	1	2	3	4
b) At any point after the surgery except during the past 6 months	0	1	2	3	4
12. Difficulty swallowing					
a) During the past 6 months	0	1	2	3	4
b) At any point after the surgery except during the past 6 months	0	1	2	3	4
13. Food getting stuck in throat during swallowing					
a) During the past 6 months	0	1	2	3	4
b) At any point after the surgery except during the past 6 months	0	1	2	3	4
14. Painful swallowing					
a) During the past 6 months	0	1	2	3	4
b) At any point after the surgery except during the past 6 months	0	1	2	3	4
15. Feeling like something is stuck in my throat					
a) During the past 6 months	0	1	2	3	4
b) At any point after the surgery except during the past 6 months	0	1	2	3	4
16. Having swollen lymph nodes in neck					
a) During the past 6 months	0	1	2	3	4
b) At any point after the surgery except during the past 6 months	0	1	2	3	4
17. Having food debris or particles in tonsils					
a) During the past 6 months	0	1	2	3	4
b) At any point after the surgery except during the past 6 months	0	1	2	3	4
18. Having bad breath					
a) During the past 6 months	0	1	2	3	4
b) At any point after the surgery except during the past 6 months	0	1	2	3	4

Questions and the associated subscales: ‘airway and breathing’ questions 1, 7, 11; ‘infection’ questions 2, 8, 9, 16; ‘health care utilization’ questions 3, 4, 5, 6; ‘eating and swallowing’ questions 12, 13, 14, 15; ‘cost of care’ question 10; ‘breath’ questions 17, 18. Each subscale is scored so that the final scores range from 0 (minimum score) to 100 (maximum score).

The questionnaire was translated into Finnish using forward and back translation, following guidelines created by Wild et al. [25]. Because TAHSI questions evaluate symptoms during the past six months, we added an additional field to every question enquiring whether the symptom had bothered the patient at any point after the surgery excluding the past six months (Table 1).

In TAHSI, each question is scored using a 5-point Likert scale, ranging from 0 (not a problem) to 4 (severe problem). For each subscale, item scores are summed to obtain a subscale raw score. The subscale raw scores are scaled to final scores that range from 0 to 100 using the following formula: scaled score = [(raw score—min score)/(max score—min score)] × 100, where max score indicates the maximum possible subscale score, and min score indicates the minimum possible subscale score. High scores indicate a high burden of disease, and low scores indicate an asymptomatic person.

In addition to TAHSI questions, we asked participants their current height and weight, smoking status, whether their tonsils had been reoperated, whether they had had a peritonsillar abscess, if they had been diagnosed with obstructive sleep apnoea, and for their overall satisfaction with the result of the tonsil surgery.

### Ethical approval

The study protocol was approved by the Helsinki University Hospital Ethics Committee (authorization number HUS/13032/2022), and an institutional research permit (§43/2022) was granted by the Department of Otorhinolaryngology, Helsinki University Hospital, Helsinki, Finland. All participants from the age of 15 years up signed their informed consent in REDCap at the time of answering the questionnaire. For participants younger than 15 years, written informed consent was obtained from the child’s caregiver via mail.

### Statistical analysis

Data was analysed using SPSS (version 28.0, IBM Corp., Amonk, NY, USA). Kolmogorov–Smirnov tests were used to assess the distribution of data. Based on these tests, our data were not normally distributed. Categorical variables were reported as frequencies and percentages, and continuous variables as medians and interquartile ranges (IQR). Mann–Whitney U tests were used to compare continuous and ordinal variables between the groups. Dichotomous data were analysed using Pearson’s chi-squared tests. A *P*-value < 0.05 was considered statistically significant.

## Results

A total of 1077 children under the age of 16 years had tonsil surgery at the study hospital between 1.1.2010 and 31.12.2011. Of these patients, 83 were excluded, mainly due to missing contact information. Recruitment letters were sent to 994 participants, and of those, 267 (26.9%) answered the survey; 87 were operated with TT and 180 with TE. All TTs (N = 87) were done because of SDB. Indications for TE were both SDB (N = 102) and infection (N = 78). Because the aim of this study was to compare the long-term effectiveness of TT in comparison with TE in the treatment of SDB, we excluded those who had TE due to infection from further analyses. Of the 994 recruited patients, 700 were operated due to SDB and the survey response rate among SDB patients was 27.0% (N = 189).

The final study population consisted of 189 respondents, of which 87 had undergone TT and 102 had undergone TE. The median follow-up was 11.8 years in the TT group and 12.4 years in the TE group. The median age in the TE group was significantly higher than in the TT group at the time of surgery (6.7 vs 5.4 years, *P* < 0.01). Current weight and height were reported by 78 (89.7%) respondents in the TT group and 97 (95.1%) in the TE group, and the median BMI was significantly lower in the TT group (*P* < 0.01). Baseline characteristics are presented in Table 2. TTs were performed by 37 surgeons and TEs by 33 surgeons, respectively. Of the TTs, 35 (40.2%) were performed by otolaryngology residents and 52 (59.8%) by specialists and of the TEs, 27 (26.5%) were performed by otolaryngology residents and 75 (73.5%) by specialists.

**Table 2:** Baseline characteristics of the study participants

	Tonsillotomy	Tonsillectomy
N (%)	87 (46)	102 (54)
Male gender (N, %)	34 (39.1)	42 (41.2)
Median age at surgery (years, range)	5.4 (2.3–13.2)	6.7 (1.9–15.8)
Median age at the time of the study (years, range)	17.3 (14.2–25.7)	19.0 (13.8–28.2)
Median follow-up (years, range)	11.8 (11.3–13.1)	12.4 (11.3–13.2)
Median BMI at the time of the study (IQR)	21.9 (20.6–24.8)^a^	23.5 (21.2–27.8)^b^
Current smoker (N, %)	5 (5.7)	10 (9.8)

^a^N = 78 (90%), ^b^N = 97 (95.1%)

### Revisits after tonsil surgery

Based on patient data, six patients (6.9%) in the TT group and one patient (1.0%) in the TE group revisited the study hospital during the 12-year follow-up period after surgery due to tonsil-related problems. In the TT group, obstructive symptoms were the reason for the visit for four patients, leading to resurgery in two of these cases due to tonsillar hypertrophy. One TT patient visited because of recurrent tonsillitis and was scheduled for tonsillectomy. One patient in the TT group visited due to a suspected peritonsillar abscess 10.5 years after the surgery but was diagnosed with acute tonsillitis. The one TE patient had a pharyngitis and lymphadenitis due to *Streptococcus pyogenes* type A 8.5 years after the surgery, which required treatment with intravenous antibiotics.

### Resurgery

In the TT group, six participants (6.9%) reported that their tonsils had been reoperated after the initial operation. One patient (1.0%) in the TE group reported this. Of the resurgeries for TT patients, three were done at the study hospital; two of these patients were reoperated with TE because tonsillar hypertrophy had caused the recurrence of SDB symptoms 2.7 and 3.4 years, respectively, after the initial surgery. The third of these TT patients had recurrent tonsillitis and was operated with TE 7.4 years after TT at the age of 12 years; five years after this TE, this patient had a peritonsillar abscess twice and went through a second TE in 2023.

The rest of the reoperations (3 TT, 1 TE) were performed elsewhere (private hospital or in another hospital) and information about these surgeries was given by the study participants via the electronic survey; two of these TT patients reported that the indication for the reoperation was tonsillar regrowth, and the final TT patient did not give details about the indication for resurgery. The one participant from the TE group requiring resurgery had been reoperated because of regrowth of the tonsils.

### Disease-specific quality of life

TAHSI questionnaire responses were analysed for 81 patients in the TT group and 101 patients in the TE group. Reoperated patients (6 TT and 1 TE) were excluded from the TAHSI analyses.

#### TAHSI scores regarding symptoms during the past six months

The subscale scaled scores were calculated for each subscale in both groups. Median scaled scores for each subscale in both groups were 0.00, meaning that the majority of participants had been asymptomatic during the past six months, and there were no statistically significant differences between the two groups. The TT group scored slightly higher in the subscale ‘airway and breathing’ (TT median 0.00 (IQR 0.0–16.67), TE median 0.00 (IQR 0.00–8.33)), but the difference was not statistically significant (*P* = 0.07).

#### TAHSI scores regarding symptoms at any point after surgery

One participant from the TE group did not answer the questions regarding symptoms at any point after surgery; thus, the analyses included 81 responses from the TT group and 100 from the TE group. The median subscale scores were 0.00 across both groups (meaning that the symptoms in question had not been a problem) for the following subscales: infection, health care utilization, eating and swallowing, cost of care, and breath. Furthermore, there were no statistically significant differences between the two groups. The scores for the ‘airway and breathing’ subscale in the TT group were higher than the scores in the TE group – TT median 8.33 (IQR 0.00–16.67) compared to the TE median 0.00 (IQR 0.00–8.33) – but the difference was not statistically significant (*P* = 0.08). When the three questions in the ‘airway and breathing’ subscale were closely examined, we found that participants in the TT group scored slightly higher for each question. The difference was greatest for the question about mouth breathing during the day. However, the differences between the scores for individual questions were not statistically significant (snoring *P*-value 0.26, apnoea *P*-value 0.20, mouth breathing *P*-value 0.15). Interestingly, three individuals in the TE group reported that they were diagnosed with obstructive sleep apnoea later in life, in contrast to none in the TT group.

### Recurrent and chronic tonsillitis after tonsil surgery

**Fig. 1:**
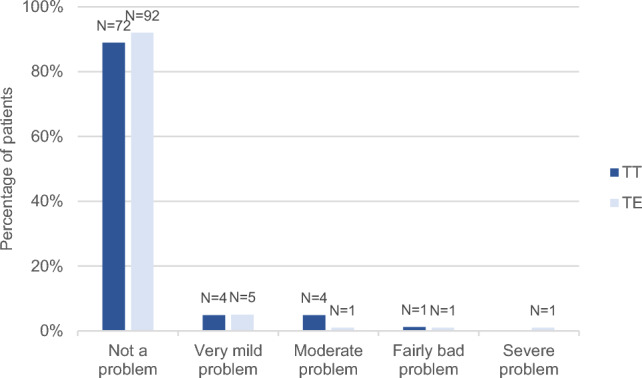
Recurrent tonsillitis at any point after the surgery except during the past six months (question 8b), tonsillotomy (TT) N = 81, tonsillectomy (TE) N = 100

**Fig. 2:**
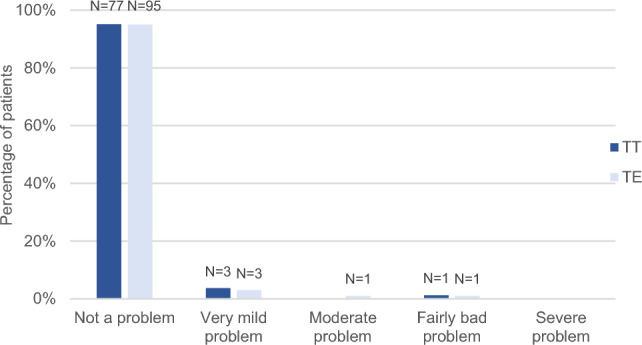
Chronic tonsillitis at any point after the surgery except during the past six months (question 9b), tonsillotomy (TT) N = 81, tonsillectomy (TE) N = 100

**Fig. 3:**
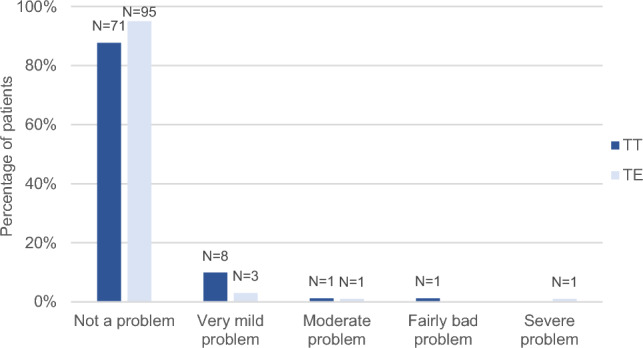
Repeated antibiotics for a sore throat at any point after the surgery except during the past six months (question 5b), tonsillotomy (TT) N = 81, tonsillectomy (TE) N = 100

There were no significant differences between the TT and TE groups regarding recurrent (*P* = 0.47) or chronic (*P* = 0.98) tonsillitis (Figs. 1 and 2). Recurrent acute infection of the tonsils had not been a problem or had only been a very mild problem at some point after surgery for 76 (93.8%) patients in the TT group and 97 (97.0%) in the TE group. In the TT group, five patients (6.2%) reported a moderate or fairly bad problem, in contrast to the TE group, in which three patients (3.0%) reported a moderate, fairly bad, or severe problem. Chronic infection of the tonsils had not been a problem or had only been a very mild problem for 80 patients (98.8%) in the TT group and 98 (98.0%) in the TE group. One participant in each group reported a fairly bad problem – 1.2% (TT) and 1.0% (TE), respectively. Concerning the question ‘Taking antibiotics over and over again for less than 2 weeks at a time for a sore throat’, patients operated with TT reported slightly worse results; for 71 (87.7%) in the TT and 95 (95.0%) in the TE group, this had not been a problem at any point after the surgery. For eight patients (9.9%) in the TT group and three (3.0%) in the TE group, it had been a very mild problem. A moderate or fairly bad problem was reported by two patients (2.5%) in the TT group, in slight contrast to the TE group, in which two (2.0%) reported moderate or severe problem. However, the difference was not statistically significant (*P* = 0.08) (Fig. 3).

### Peritonsillar abscess

None of the participants in the TT group had been diagnosed with a peritonsillar abscess at any point after surgery, while three (2.9%) patients in the TE group reported that they had had a peritonsillar abscess. As mentioned in subsection 3.2, one of these patients was initially operated with TT, then underwent TE for repeated tonsillitis and had peritonsillar abscess twice five years after TE.

### Satisfaction with the result of tonsil surgery

**Fig. 4:**
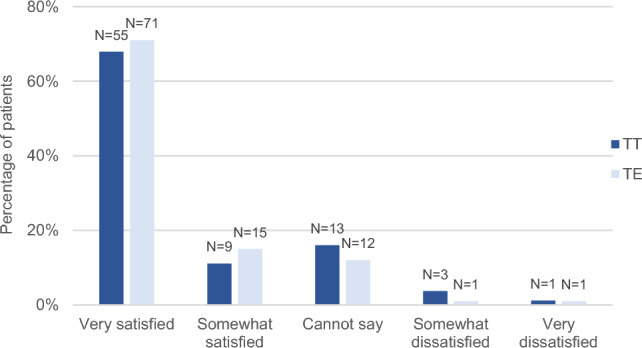
Satisfaction with the result of the tonsil surgery, median 12.1 years post operation (range 11.3–13.2), tonsillotomy (TT) N = 81, tonsillectomy (TE) N = 100

The majority of participants were satisfied with the result of surgery, and there were no statistically significant differences between the groups in this respect (*P* = 0.48) (Fig. 4). Sixty-four respondents in the TT group (79.0%) and 86 in the TE group (86.9%) were very or somewhat satisfied. Only three respondents (3.7%) in the TT group and one (1.0%) in the TE group were somewhat dissatisfied, and one person in each group was very dissatisfied. One person in the TE group did not answer this question.

## Discussion

While multiple studies have reported the safety of TT and its benefits for short-term recovery [7–11], there is a lack of publications dealing with long-term results after an operation. We assessed the long-term effectiveness of TT on disease-specific QOL, possible recurrence of tonsil-related symptoms, and rate of reoperation over a median follow-up period of 12 years. Our study presents the longest follow-up period after TT in the literature to date.

In this study, the long-term effectiveness of TT on disease-specific QOL was equal to that of TE. TAHSI questionnaire results revealed that the vast majority of participants had been asymptomatic postoperatively, and there were no significant differences between the two surgical groups in any of the subscales dealing with snoring, infections, eating difficulties, halitosis and tonsil stones, health care utilization, or cost of care. Our results are in line with the prospective study of Wireklint et al., in which TT and TE yielded equally good long-term results for health-related QOL (SF-36) six years after surgery in young adults [26]. Other long-term studies comparing TT and TE in the treatment of SDB have utilized self-constructed questionnaires rather than validated QOL tools [27, 28]. A prospective, randomized study by Hultcrantz et al. on 41 children operated with TT or TE for SDB found no significant differences between the groups with respect to general health, snoring, sleep apnoea, eating difficulties, or infections six years after surgery [28].

In this study with a rather small number of patients (N = 87), 6.9% patients operated with TT underwent resurgery, mostly because of tonsillar regrowth. In our previous study of 3141 children, we determined the rate of resurgery after TT at the study hospital, and the incidence of reoperation was 1.9% [29]. A retrospective register-based cohort study in Sweden with more than 27,000 patients reported a revision surgery rate of 3.9% after TT [30]. In both studies, the majority of the resurgeries were done due to recurrent obstructive symptoms, and surgery at a younger age was strongly associated with risk of reoperation [29, 30]. When TTs began at the study hospital in 2009, only the protruding part of the tonsil was dissected along the plane of pharyngeal pillars. Thereafter, studies revealed that the risk of postoperative haemorrhage is very low despite the technique and the depth of the removed tonsillar tissue, and today, tonsillar tissue is also removed from the tonsillar fossa [11]. Earlier, more superficial surgical techniques might explain the higher incidence of resurgery in the Swedish cohort and in our current study [30, 31]. In intracapsular tonsillectomy (ICT), the majority of the tonsillar tissue is removed down to the capsule [32], which might lower the rate of reoperation [17]. However, there are no current studies proving the superiority of ICT over TT. In a recent meta-analysis, the resurgery rate after coblation ICT was 1.4% (N = 7720), and a large study reported the resurgery rate after microdebrider ICT to be 0.12% (N = 1456) [33, 34].

During the 12-year postoperative period, four patients in the TT group revisited the study hospital due to obstructive symptoms (4.6% of the patients operated with TT), and of these, two were scheduled for reoperation. Patients operated with TT reported slightly more symptoms in the ‘airway and breathing’ section of TAHSI compared to the TE group, but the difference was not statistically significant. While we did not perform clinical evaluation on patients at the time of the study, the contribution of tonsil remnants and possible regrowth to slightly higher ‘airway and breathing’ subscale scores in the TT group remains unclear. No participants in the TT group were diagnosed with obstructive sleep apnoea, in contrast to three participants in the TE group. The median BMI was higher in the TE group, which might explain the higher reported rate of sleep apnoea. However, 10.3% of participants in the TT group did not report their current weight and height, so there might be a substantial bias in the BMI values. All in all, scores for the ‘airway and breathing’ section were low in both groups, indicating that SDB symptoms were not bothersome.

In our study, the vast majority of the TT patients had no problems with recurrent or chronic tonsillitis after the operation, and the results were similar to those for the TE group. The reported usage of antibiotics for sore throats did not differ significantly between the groups. Comparable results were obtained in two prospective studies with follow-up periods of three and six years, respectively; upper respiratory tract infections treated with antibiotics occurred to the same extent after TT and TE [28, 35]. In a randomized study with young adults operated with TT or TE due to SDB and recurrent tonsillitis, there were no differences between the groups regarding upper respiratory tract infections during a follow-up period of six years [26]. Because tonsillitis seems not to be a problem after TT, there has been growing interest in expanding the indications of tonsillotomy for recurrent and chronic tonsillitis, as well as periodic fever, aphthous stomatitis, pharyngitis, and cervical adenitis (PFAPA) syndrome [36–38].

It has been speculated that after TT, remaining tonsillar tissue becomes scarred, leaving a patient predisposed to peritonsillar abscess formation. During the 12-year study period covered here, no patient in the TT group was diagnosed with a peritonsillar abscess, in contrast to three patients in the TE group. Corresponding results were obtained in a retrospective chart review by Soaper et al. in which no significant difference in the rate of peritonsillar abscess formation was noted after ICT compared to TE over a follow-up period of up to 14 years (mean 8.2 years) [34]. Peritonsillar abscess formation after TE might be due to infection of the minor salivary glands [39]. In histological studies of regrown tonsils after TT, no signs of scarring have been detected [16, 29]. According to our study, TT does not seem to predispose a patient to peritonsillar abscess formation, but an even longer follow-up might be required to confirm these results, as the highest incidence of peritonsillar abscesses occurs between 15 to 30 years of age [40].

The majority of the patients reported to be very satisfied or quite satisfied with the result of tonsil surgery: 79% in the TT group and 86% in the TE group, respectively. Only four patients in the TT group and two patients in the TE group were somewhat or very dissatisfied. Similarly, in a prospective study over six years, young adults reported high satisfaction with the results of TT and TE, with no significant differences between the two groups [26]. Patients operated with appropriate indications seem to be satisfied and benefit from tonsil surgery. Both TT and TE have been shown to improve QOL of children, simultaneously reducing indirect healthcare costs [14, 41].

The strength of our study is the long follow-up period after tonsil surgery; symptoms most often recur or problems occur in the 10-year period following tonsil surgery. However, the retrospective study design we used is a limitation. The survey response rate was fairly low (27%), which is a common phenomenon seen with questionnaire studies. A limitation of this study might be that only the patients who were satisfied replied to the questionnaire. The QOL instrument TAHSI is not validated for adults, but according to our knowledge there is no other disease-specific QOL instrument for adults that would cover both obstructive and infectious symptoms. TOI-14 is another tonsillar-disease-specific QOL instrument for adults, but it only covers symptoms of chronic tonsillitis [42]. Because the indication of tonsil surgery was SDB, we wanted to include both obstructive and infective symptoms when assessing disease-specific QOL after TT and, therefore, TAHSI was chosen.

## Conclusion

Long-term clinical effectiveness of TT seems to be excellent. Disease-specific QOL after tonsil surgery showed no statistically significant differences between TT and TE, and the majority of patients in both groups were very satisfied with the result of tonsil surgery. TT was not associated with increased risk of throat infections in comparison to TE over a median follow-up period of 12 years. The vast majority of TT patients had been asymptomatic after surgery, and only small number of patients required a revisit or reoperation due to tonsil-related problems. These promising long-term results in combination with advantages of TT in terms of lower postoperative morbidity reinforce the superiority of TT over TE in the treatment of children’s SDB caused by tonsillar hyperplasia. Additional long-term studies are needed to confirm our results.

## Data Availability

The data of this study are available from the corresponding author, Julia Virkkunen, upon reasonable request.
